# The Complete Genome Sequence of *Sarcoramphus papa* (Cathartidae), the King Vulture

**DOI:** 10.56179/001c.68132

**Published:** 2023-01-21

**Authors:** Therese A Catanach, Stacy Pirro

**Affiliations:** 1 Department of Ornithology, Academy of Natural Sciences, Drexel University,; 2 Biodiversity, Iridian Genomes

**Keywords:** genome, bird, vulture

## Abstract

*Sarcoramphus papa* is a New World Vulture found predominantly in tropical lowland forests stretching from southern Mexico to northern Argentina. We present the whole genome sequence of this species. Illumina paired-end reads were assembled by a de novo method followed by a finishing step. The raw and assembled data are publicly available via GenBank: Sequence Read Archive (SRR19167646) and assembled genome (GCA_027580115).

## Introduction

The New World Vulture *Sarcoramphus papa* is one of the largest birds in the Neotropics. Adults are predominantly white with black flight and tail feathers. The head and neck are bald, and the skin is extremely colorful and can include yellow, orange, blue, purple, and red markings that contrast sharply with the white eyes. Adults also have a very noticeable orange fleshy caruncle above the cere. Juveniles are sooty black and take several years to develop adult plumage and caruncles.

## Methods

A single museum specimen from Museum of Southwestern Biology (MSB:Bird:27868) was used for this study. DNA extraction was performed using the Qiagen DNAeasy genomic extraction kit using the standard process. A paired-end sequencing library was constructed using the Illumina TruSeq kit, according to the manufacturer’s instructions. The library was sequenced on an Illumina Hi-Seq platform in paired-end, 2 × 150bp format. The resulting fastq files were trimmed of adapter/primer sequence and low-quality regions with Trimmomatic v0.33 (Bolger, Lohse, and Usadel 2014). The trimmed sequence was assembled by SPAdes v2.5 (Bankevich et al. 2012) followed by a finishing step using Zanfona v1.0 (Kieras 2021) to make additional contig joins based on conserved regions in related species.

## Results

The genome assembly yielded a total sequence length of 1,227,506,220 bp over 14,173 scaffolds.

## Data Availability

Raw and assembled data is publicly available via GenBank:
